# Anti-carcinogenic effects of exercise-conditioned human serum: evidence, relevance and opportunities

**DOI:** 10.1007/s00421-021-04680-x

**Published:** 2021-04-17

**Authors:** Richard S. Metcalfe, Rachael Kemp, Shane M. Heffernan, Rachel Churm, Yung-Chih Chen, José S. Ruffino, Gillian E. Conway, Giusy Tornillo, Samuel T. Orange

**Affiliations:** 1 Applied Sports, Technology, Exercise and Medicine (A-STEM) Research Centre, College of Engineering, Swansea University, Swansea, SA1 8EN Wales UK; 2 Department of Physical Education, National Taiwan Normal University, Taipei, Taiwan; 3 University of Bristol, Bristol, UK; 4 In Vitro Toxicology Group, Institute of Life Sciences, College of Medicine, Swansea University, Swansea, UK; 5 European Cancer Stem Cell Research Institute, School of Biosciences, Cardiff University, Cardiff, UK; 6 School of Biomedical, Nutritional and Sport Sciences, Faculty of Medical Sciences, Newcastle University Centre for Cancer, Newcastle University, Newcastle upon Tyne, UK

**Keywords:** Exercise, Physical activity, Cancer prevention, Cancer therapy, Cancer cell growth, Cancer cell proliferation, Cancer cell apoptosis, Exercise-conditioned serum

## Abstract

Regular physical activity reduces the risk of several site-specific cancers in humans and suppresses tumour growth in animal models. The mechanisms through which exercise reduces tumour growth remain incompletely understood, but an intriguing and accumulating body of evidence suggests that the incubation of cancer cells with post-exercise serum can have powerful effects on key hallmarks of cancer cell behaviour in vitro. This suggests that exercise can impact tumour biology through direct changes in circulating proteins, RNA molecules and metabolites. Here, we provide a comprehensive narrative overview of what is known about the effects of exercise-conditioned sera on in vitro cancer cell behaviour. In doing so, we consider the key limitations of the current body of literature, both from the perspective of exercise physiology and cancer biology, and we discuss the potential in vivo physiological relevance of these findings. We propose key opportunities for future research in an area that has the potential to identify key anti-oncogenic protein targets and optimise physical activity recommendations for cancer prevention, treatment and survivorship.

## Introduction and overview

Cancer incidence is high worldwide, with an estimated 19.3 million new cancer cases and almost 10 million cancer deaths in 2020 (Sung et al. 2021). It is estimated that the global cancer burden will increase to 28.4 million cases in 2040 (Sung et al. 2021). Female breast cancer is now the most commonly diagnosed cancer globally, accounting for 11.7% of all new cases, followed by lung (11.4%), colorectal (10.0%), and prostate (7.3%) cancers (Sung et al. 2021). Cancer incidence rates rise uniformly with increasing Human Development Index (a quantitative indicator of a countries development status); the incident rate for all cancers is around 2.5 times higher in the most developed versus the least developed countries (Bray et al. 2018; Sung et al. 2021). The increase in morbidity and premature death related to cancer incidence exerts a substantial human cost and places considerable pressure on healthcare systems (Torre et al. 2016). Whilst the aetiology of cancer is complex, there is a modifiable component to cancer risk and outcome, with estimates that as many as 40% of all cancers could be prevented through alteration of lifestyle risk factors at the population level, including an increase in physical activity (Wilson et al. 2018). Indeed, regular physical activity is a central tenet of international cancer prevention recommendations (Rock et al. 2020). Moreover, regular physical activity *after* cancer diagnosis is associated with a reduced risk of tumour recurrence and mortality in patients with colon, breast or prostate cancer, supporting a role for physical activity as an adjunct cancer therapy (Ballard-Barbash et al. 2012).

At present, the physiological and molecular mechanisms underlying the beneficial impact of physical activity on cancer risk and outcome are largely unclear. Part of the effect may be driven indirectly through the positive impact of physical activity on body mass and composition; indeed, many endogenous cancer risk factors are strongly associated with adiposity, including insulin resistance and circulating concentrations of sex hormones, insulin-like growth factors and inflammatory cytokines (McTiernan 2008). However, there is also accumulating evidence that exercise and physical activity can promote physiological changes that directly alter tumour growth and behaviour (Christensen et al. 2018). This includes improvements in immune function and immune surveillance (Idorn and Hojman, 2016), but emerging evidence also suggests that other serological changes elicited by exercise can also be anti-carcinogenic, as evidenced by alterations in in vitro cancer cell growth and behaviour following incubation with post-exercise serum (Hojman et al. 2011; Orange et al. 2020). These findings suggest that physical activity/exercise can promote the release of metabolites, proteins or RNA species with anti-tumorigenic properties. Furthering our understanding of these effects has the potential to isolate novel therapeutic targets and to enhance our ability to develop specific physical activity recommendations for cancer prevention, treatment and survivorship.

The purpose of this narrative review is to summarise the current state of evidence for the effects of exercise-conditioned serum on cancer cell behaviour in vitro. In doing so, we consider the key limitations of the current body of literature, both from the perspectives of exercise physiology and in vitro cancer biology, and we also discuss the potential in vivo physiological relevance of these findings. In addition, throughout the review, we highlight key opportunities and provide a comprehensive roadmap for future research to improve our understanding of the beneficial effects of exercise on the prevention and treatment of cancer.

## Physical activity, exercise and cancer: epidemiological evidence

Strong epidemiological evidence suggests that people who are regularly physically active have a lower risk of developing common cancer types. The 2018 Physical Activity Guidelines Advisory Committee (PAGAC) Scientific Report concluded that achieving the highest versus lowest level of physical activity reduces the risk of developing seven different cancers; namely colon, breast, kidney, endometrium, bladder, and stomach cancer, and oesophageal adenocarcinoma (Physical Activity Guidelines Advisory Committee 2018). Relative risk reductions ranged from approximately 10% to 20% in magnitude (McTiernan et al. 2019). These conclusions were supported by a 2018 review by the American College of Sports Medicine (ACSM) Roundtable, which further determined that physical activity also protects against liver cancer (Patel et al. 2019). Moreover, the ACSM Roundtable found moderate associations between greater amounts of post-diagnosis physical activity and decreased cancer-specific mortality in colon, breast, and prostate cancers, with a relative risk reduction of approximately 30% (Patel et al. 2019). The World Cancer Research Fund/American Institute for Cancer Research (WCRF/AICR) 2018 Continuous Update Project concluded that the evidence for a protective effect of physical activity was strong for colon, postmenopausal breast, and endometrial cancers, and was limited but suggestive for premenopausal breast, oesophageal, lung, and liver cancers (World Cancer Research Fund/American Institute for Cancer Research 2018). Therefore, whilst there are some discrepancies between reports regarding the strength of evidence for specific cancer sites, the epidemiological data are clearly supportive for physical activity as a strategy to reduce incident cancer, particularly for colon, breast and endometrial cancers.

It is noteworthy that almost all of the epidemiological evidence relates to leisure-time (i.e. active recreation, structured exercise and sports) and occupational aerobic physical activity (Physical Activity Guidelines Advisory Committee 2018; World Cancer Research Fund/American Institute for Cancer Research 2018). There is much less data on the association between cancer risk and physical activity in the household and transport domains. Similarly, despite accumulating evidence supporting a role for resistance exercise in cancer survivorship (Schmitz et al. 2019), and the fact that resistance exercise is recommended for overall health (Physical Activity Guidelines Advisory Committee Scientific Report 2018; Garber et al. 2011), there is a lack of epidemiological data for this type of activity in relation to cancer incidence. This paucity of evidence is highlighted in physical activity guidelines for cancer prevention, with the American Cancer Society (ACS) recommending that adults should engage in 150–300 min/week of moderate-intensity or 75–150 min/week of vigorous-intensity aerobic physical activity (or an equivalent combination of both), with less specific instructions for resistance-type exercise (‘some muscle‐strengthening activity at least 2 days each week’) (Rock et al. 2020).

The best available evidence suggests that there is a dose–response relationship between the amount of physical activity and the risk of several cancer types. A recent pooled analysis of data from over 750,000 adults followed for 10 years showed linear dose–response associations between physical activity and incidence of breast, colon, and endometrial cancer, oesophageal adenocarcinoma, and head and neck cancer (Matthews et al. 2019). For these cancer types, engaging in physical activity above the recommended amount (i.e. > 300 min·wk^−1^ of moderate-intensity physical activity) was associated with additional risk reduction (Matthews et al. 2019). Importantly, there does not appear to be a threshold of physical activity below which there is no cancer-preventive benefit, suggesting that any level of physical activity is better than none (Mctiernan et al. 2019); however, the magnitude of the reduction in cancer risk for the same ‘dose’ of physical activity does appear to be less pronounced compared with cardiovascular and metabolic disease (Rock et al. 2020).

Despite increasing efforts to shape the dose–response curve between physical activity dose and cancer incidence (Physical Activity Guidelines Advisory Committee 2018; World Cancer Research Fund/American Institute for Cancer Research, 2018; Matthews et al. 2019), the influence of exercise intensity remains unclear. The totality of epidemiological evidence suggests that moderate-to-vigorous-intensity physical activity is inversely associated with several cancer types (Patel et al. 2019; Rock et al. 2020); however, the evidence relating to the independent effects of light- moderate-, and vigorous-intensity physical activity is limited, which is, at least, partly due to the wide range of methods used to classify intensity of physical activity, and the challenges in accurately demarcating intensity domains on a large scale. In a subset analysis of 309,881 adults, Matthews et al. (2019) reported overall negative associations with risk of breast and kidney cancer for moderate-intensity activity, while vigorous-intensity activity was associated with reduced risk of endometrial cancer. However, there was large uncertainty around the estimates, highlighting that more evidence is needed to better understand the optimal intensity of physical activity for cancer risk reduction.

In summary, there is strong epidemiological evidence that regular aerobic physical activity in the leisure-time or occupational domain protects against several cancer types, mostly in a dose–response manner. Aside from the importance of these observations from the perspective of exercise prescription and promotion, and public health, they have also stimulated substantial research interest towards exploring the physiologically relevant effects of exercise that impact upon tumour genesis, proliferation and metastasis (Ashcraft et al. 2016; Eschke et al. 2019).

## Putative biological mechanisms

The prevailing hypothesis with regard to the mechanisms underlying the anti-oncogenic effects of physical activity focuses on its interactions with energy balance and adiposity. Excess body fatness resulting from a positive energy balance increases insulin resistance and chronic low-grade inflammation, which are known to be important risk factors for cancer (McTiernan 2008). Raised fasting and postprandial circulating insulin increases the bioavailability of insulin-like growth factor 1 (IGF-1) through the inhibition of hepatic IGF binding protein 1 (IGFBP-1) and IGFBP-2 secretion (Brismar et al. 1994), and increases hepatic synthesis of IGF-1 through the upregulation of growth hormone receptors (Leung et al. 2000). Circulating IGF-1 is positively related to several cancer types (Knuppel et al. 2020), and is known to stimulate healthy and malignant cell proliferation through ligand binding to the IGF-1 receptor and activation of downstream intracellular pathways, including the PI3K-Akt-mTOR and mitogen-activated protein kinase (MAPK) pathways (Iams and Lovly 2015). Chronic low-grade inflammation induced by excess adiposity, characterised by macrophage infiltration and secretion of pro-inflammatory cytokines, can promote DNA damage and genomic instability through the induction of reactive oxygen and nitrogen species (Coussens and Werb 2002). Pro-inflammatory cytokines, such as tumour necrosis factor alpha (TNF- α) and interleukin-1 beta (IL-1β), also stimulate cell proliferation and invasive capacity of pre-malignant cells, causing a shift to a neoplastic phenotype (Baird et al. 2016; Esquivel-Velázquez et al. 2015). Other putative biological mechanisms related to excess body fat include enhanced circulating leptin, which is a growth factor in normal and malignant cells, mainly through JAK/STAT signalling (Hu et al. 2002), and increased sex steroid hormones. Excess body fat promotes the conversion of androgen precursors to oestrogen through enhanced aromatase activity in the adipose tissue, which may increase the risk of postmenopausal, oestrogen-receptor-positive breast cancer (Gérard and Brown 2018). Similarly, obesity is linked to lower levels of serum sex hormone-binding globulin (Cooper et al. 2015), which may increase the risk of hormone-sensitive cancers, such as prostate and postmenopausal breast cancer, by increasing exposure to bioavailable testosterone and oestrogen, respectively.

Physical activity energy expenditure largely explains variations in total daily energy expenditure (Thompson et al. 2009, 2015) and, for this reason, is known to be important for long-term weight maintenance, as well as the avoidance of weight regain after weight loss (Donnelly et al. 2009). Thus, much of the protective effect of physical activity on cancer risk has been ascribed to indirect, adiposity-dependent mechanisms (described above) (McTiernan 2008). However, recent epidemiological evidence shows that physical activity is associated with a reduced risk of common cancers independently of body mass index, with the exception of endometrial cancer (Matthews et al. 2019; Moore et al. 2016). A recent Mendelian randomisation study using UK Biobank data also showed that physical activity was inversely associated with colorectal and breast cancers independent of adiposity (Papadimitriou et al. 2020). Furthermore, a meta-analysis of randomised pre-clinical studies showed that exercise training alone (i.e. without dietary modification) suppresses tumour growth in rodents (Eschke et al. 2019). As such, there is a growing appreciation that the plethora of physiological responses to both acute and chronic physical activity and exercise may exert *direct* anti-tumorigenic effects (Christensen et al. 2018). This includes the mobilisation and redistribution of key immune-cell populations (e.g., natural killer T-cells) (Idorn and Hojman 2016). However, an intriguing and accumulating body of evidence also suggests that incubation of cancer cells with exercise-conditioned serum (i.e. no immune cells) can also have powerful effects on key hallmarks of cancer cell behaviour (Orange et al. 2020). This suggests that exercise can have an impact on tumour biology through direct changes in the circulating transcriptome/metabolome/proteome and opens up a new and exciting area of research, with the potential to isolate key anti-oncogenic serological targets and to enhance our ability to develop optimal physical activity recommendations for cancer prevention, treatment and survivorship.

## Anti-carcinogenic effects of exercise-conditioned human serum

### Effects of acute exercise

**Fig. 1:**
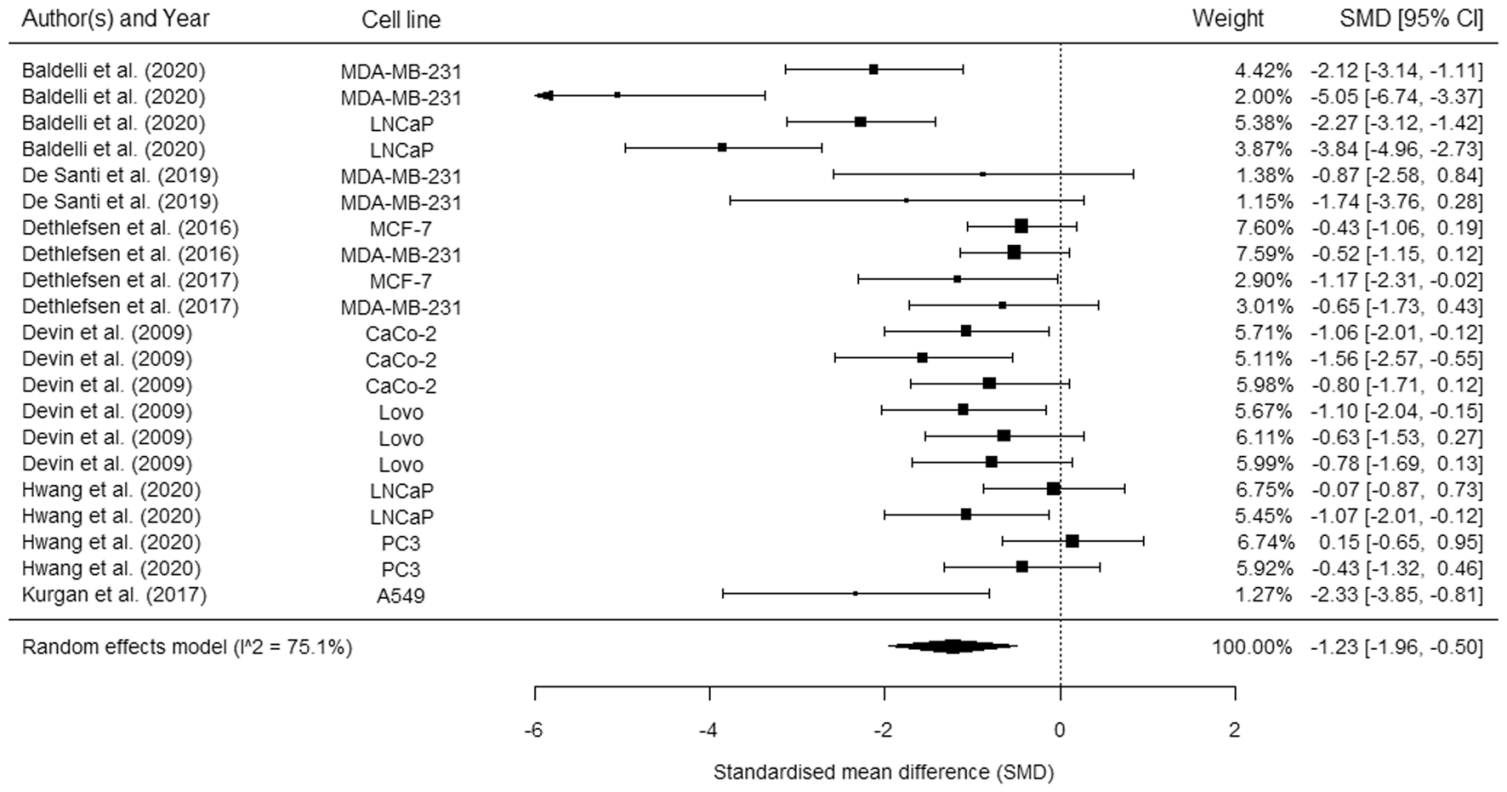
Forest plot showing the pooled effect of serum collected immediately post exercise on cancer cell proliferation. Figure reproduced from Orange et al. (2021), distributed under an open access Creative Common CC BY license

Exercise is a subcomponent of physical activity encompassing planned, structured activities purposefully carried out to sustain or improve physical fitness (Caspersen et al. 1985; Dasso 2019). The onset of exercise results in a myriad of transient changes in serum that are dependent on relative exercise intensity, exercise duration and exercise mode (Contrepois et al. 2020; Guseh et al. 2020). One of the key hallmarks of cancerous cells is increased cell survival or viability (Ruiz-Casado et al. 2017); over the last decade, evidence has accumulated to suggest that the systemic alterations induced by exercise can have direct effects on cancer cell viability in vitro. This was first demonstrated by Rundqvist et al. (2013) who incubated the human prostate cancer cell line, LNCaP, with serum collected from young healthy males before and 2 h after a 65 min bout of moderate-intensity aerobic exercise (50–65% V̇O_2max_). The number of viable prostate cancer cells following a 96 h incubation with post-exercise serum in vitro was reduced by ~ 30% when compared with serum collected pre-exercise (Rundqvist et al. 2013). This finding has since been replicated using LNCaP cells and sera derived from a comparable population (young healthy men) (Baldelli et al. 2020), and it has also demonstrated in human breast cancer cell lines using sera derived from young healthy women and women with breast cancer (Baldelli et al. 2020; Dethlefsen et al. 2016, 2017a), human colon cancer cell lines using sera derived from colon cancer survivors (Devin et al. 2019), and human lung cancer cells using sera derived from healthy men (Kurgan et al. 2017). Indeed, Orange et al. (2020) recently summarised studies that had examined the effect of serum collected immediately post-exercise on in vitro cancer cell viability and, in a meta-analysis, reported an 8.6% decrease in cell viability compared with serum collected pre-exercise (Fig. 1). Importantly, there is no evidence that exercise-conditioned serum has an effect on growth in non-malignant ‘control’ cell lines, suggesting that the serological effects of exercise are suppressive specifically in cancer cells that harbour aberrant activation of signalling pathways (Kurgan et al. 2017; Rundqvist et al. 2013). In addition, the short-term effects on cancer cell viability have been shown to translate into a strong reduction (30–80%) in longer term ‘clonogenic potential’, that is the ability of the cancer cells to grow and form new colonies, which is considered the most robust in vitro measure of cell growth (Baldelli et al. 2020; Kurgan et al. 2017). Moreover, mice inoculated with cancer cells conditioned in (human) post-exercise sera subsequently developed less tumours compared with mice injected with cells conditioned in pre-exercise serum (Dethlefsen et al. 2017a).

The anti-proliferative effect of post-exercise serum on cancer cells could result from a reduction in the rate of cell proliferation, the induction of cell death (e.g., apoptosis), or a combination of the two. Further, the relative contribution or importance of each of these mechanisms could change depending on the time point post-exercise, the cancer cell type, the serum donor population, and the characteristics of the exercise bout. A very limited number of studies have examined this using appropriate methods in humans; however, it has tentatively been suggested that acute-exercise-conditioned serum does not affect cancer cell apoptosis, suggesting that the growth-inhibitory effect is (largely) mediated through a suppression of proliferative signalling pathways (Devin et al. 2019; Rundqvist et al. 2013). This is somewhat surprising since post-exercise increases in serum concentrations of myokines (skeletal muscle-derived cytokines/peptides), such as interleukin (IL)-6, have previously been associated with cancer cell apoptosis in vitro (Bharti et al. 2016; Liu et al. 2010). Yet, consideration should be given to the timing of post-exercise sample collection, as demonstrated by a study showing that exercise-conditioned sera from a mouse model induces cancer cell apoptosis through an increase in caspase activity immediately, but not 2 h, post-exercise (Hojman et al. 2011). Only one of the aforementioned human studies measured apoptosis after applying human serum collected immediately post-exercise (Devin et al. 2019), whilst both studies examined serum collected 2 h post-exercise (Devin et al. 2019; Rundqvist et al. 2013).

It is also currently unclear how long the suppressive effect on cancer cell growth persists after exercise because studies have either only collected samples at one time point post exercise (Hwang et al. 2020; Rundqvist et al. 2013) or, where multiple samples have been collected, they have not been collected at consistent time points across studies (Baldelli et al. 2020; Dethlefsen et al. 2016, 2017a; Devin et al. 2019; Kurgan et al. 2017). In some cases, the effect has been short-lived and lost in serum collected 2 h post exercise (Devin et al. 2019), whilst others have been able to detect an effect of serum for up to 24 h post exercise (Baldelli et al. 2020; Kurgan et al. 2017) (Table 1). Future research in this area should carefully map the time course of the effects of exercise-conditioned sera on cell proliferation and cell death in a range of cancer cell types with different genetic profiles, following a range of exercise bouts, using serum from various populations with detailed health status.

**Table 1:** Map of post-exercise effects on cancer cell viability / growth

Study	Participants	Cancer cell line	Exercise bout	Post-exercise time point				
				0	1-h	2-h	4-h	24-h
Baldelli et al. (2020)	Young and healthy males and females (*n* = 30)	LNCaP (prostate) and MDA-MB-231 (breast)	Cycling. Progressive aerobic (4 × 5-min at 50%, 55%, 60%, 70% *W*max) and HIIE (10 × 90-s at 90% *W*max interspersed with 180-s at 55% *W*max) followed by exercise to exhaustion at 90% *W*max	↓			↓	↓
Dethlefsen et al. (2016)	Middle-aged women with breast cancer (*n* = 20)	MCF-7 and MDA-MB-231 (breast)	30 min warm up followed by 60 min of whole-body resistance exercise and 30 min of HIIE cycling (80–85% HRmax). No other details provided	↓				
Dethlefsen et al. (2017a )	Young healthy females (*n* = 7)	MCF-7 and MDA-MB-231 (breast)	Moderate intensity continuous cycling (2-h at 55% VO_2_max)	↓				
Devin et al. (2019)	Male colon cancer survivors (*n* = 10)	CaCO-2 and LoVo (colon)	HIIE cycling (4 × 4 min at 85–95% HRmax)	↓		↔		
Hwang et al. (2020)	Healthy young males (*n* = 12)	PC3 and LNCaP (prostate)	Moderate intensity continuous cycling (20-min at 50% VO_2_max followed by 45-min at 65% VO_2_max)	↓				
	Healthy older males (*n* = 12)	PC3 and LNCaP (prostate)	Moderate intensity continuous cycling (20-min at 50% VO_2_max followed by 45-min at 65% VO_2_max)	↔				
Kurgan et al. (2017)	Healthy young males (*n* = 6)	A549 (lung)	HIIE cycling (6 × 1-min at 90% *W*max)	↓	↓			↓
Rundqvist et al. (2013)	Young healthy males (*n* = 10)	LNCaP (colon)	Moderate intensity continuous cycling (20-min at 50% VO_2_max followed by 40-min at 65% VO_2_max)			↓		

*W*_max_ maximal power output achieved during an incremental exercise test to volitional exhaustion, *HIIE* High-intensity interval exercise, *HR*_max_ maximal heart rate

↓Decreased cancer cell viability/growth; ↔ , no change in cancer cell viability /growth

Although these studies provide preliminary evidence that acute exercise-conditioned serum suppresses cancer cell growth, they should be interpreted with some caution as there are also important methodological limitations. First, they almost exclusively make inferences based on a comparison of pre- (i.e. inactive) compared to post-exercise serum and do not include a time-matched resting control condition (Baldelli et al. 2020; Devin et al. 2019; Hwang et al. 2020; Kurgan et al. 2017; Rundqvist et al. 2013). On the face of it, a comparison of pre- and post-exercise serum may seem like a reasonable study design, but it means that the anti-oncogenic effects cannot be specifically attributed to exercise alone. Indeed, the serum profile may be subject to circadian fluctuation over time (Carroll et al. 2019), and repeated venous blood sampling (used in some studies) may result in physiological changes that could also have anti-tumorigenic effects, for example an increase in circulating adrenaline (Carruthers et al. 1970). Indeed, plasma cytokines have been shown to increase from pre- to post-acute aerobic exercise, but this increase was similar in magnitude and not statistically different from a time-matched control condition (Windsor et al. 2018). Only Dethlefsen et al. (2017a) included a separate resting control condition within their study design; however, they did not collect a ‘pre’ sample in either the resting or the exercise conditions, meaning that day-to-day variation in the serological profile could confound their analysis. Taken together, there is a clear need for further studies that include a time-matched inactive control condition, in a randomised, crossover design, so that the effects of acute exercise can be isolated. It is also worth highlighting that some studies have made inferences based on comparisons of sera pooled from all individuals pre- and post-exercise and this approach may mask important inter-individual differences in response (Rundqvist et al. 2013).

It should also be noted that there is a high level of variability in the reported effects of acute-exercise-conditioned serum on in vitro cancer cell viability in studies conducted to date (Orange et al. 2020). This is perhaps unsurprising given the diverse range of populations, cancer cell lines, and exercise characteristics that have been included across different independent studies, all of which could be hypothesised to alter the presence and/or magnitude of the effect of exercise-conditioned serum on growth/viability. Interestingly, in the meta-analysis by Orange et al. (2020), covarying for different cancer types (breast vs other), disease status (healthy vs cancer survivor), or exercise mode (continuous vs interval training) did not alter the effect magnitude or heterogeneity. However, these findings should be treated with some caution given the small number of studies included and the fact that this analysis was largely based on comparisons of effect sizes *between* independent studies (i.e. with different methodologies) rather than those reported *within* the same studies (i.e. where methodologies are standardised). As an example of the potential for factors, such as participant characteristics, to have an impact on the effect of exercise-conditioned serum on cancer cell behaviour, one study reported an anti-growth effect of post-exercise serum from older (mean age 63 years) but not younger (mean age 28 years) men in prostate cancer cells following 60 min of moderate-intensity cycling (Hwang et al. 2020).

The mode, intensity and duration of exercise is known to have a key impact on the serological response during exercise and in the post-exercise recovery period (Metcalfe et al. 2015; Stokes et al. 2013; Williams et al. 2013). For this reason, it can be speculated that different modes, durations and intensities of exercise will also differentially affect the anti-oncogenic effects of acute-exercise-conditioned serum. A significant serum-stimulated reduction in human cancer cell viability / growth has been demonstrated using several different acute exercise protocols (Table 1), including moderate-intensity steady-state aerobic exercise (Dethlefsen et al. 2017a; Hwang et al. 2020; Rundqvist et al. 2013), high-intensity aerobic interval exercise (HIIE) (Devin et al. 2019; Kurgan et al. 2017), a combined session of HIIE and whole-body resistance exercise (Dethlefsen et al. 2016), and a combined incremental aerobic and HIIE session (Baldelli et al. 2020). Yet, with the exception of Dethlefsen et al. (2017a) who showed a similar reduction in breast cancer cell growth after both 1- and 2-h of moderate-intensity cycling, no single study has made a direct comparison of the effects of different modes, intensities, or durations of acute exercise within the same participants, which makes inferences on the effect of characteristics challenging. There is a clear need for this knowledge gap to be addressed by future research studies in this area. It is also interesting to note that the majority of the exercise bouts have involved prolonged exercise durations (1–2 h) (Dethlefsen et al. 2016, 2017a; Hwang et al. 2020; Rundqvist et al. 2013) or, in some cases, exercise to the point of volitional exhaustion (Baldelli et al. 2020). It is currently unknown whether moderate-intensity aerobic exercise of a shorter duration (e.g. bouts of 10–30 min), or acute resistance exercise alone, also have a growth-inhibitory effect on cancer cells. These are important questions because prolonged moderate-intensity aerobic exercise lasting > 60 min or exercise of a vigorous intensity may not be feasible for some cancer survivors or those with the highest risk of developing common cancers, such as older, overweight adults with multimorbidity. Establishing the impact of different modes, durations and intensities of exercise on the anti-carcinogenic effects of exercise-conditioned serum will be of key importance to the optimisation of exercise recommendations for cancer prevention and treatment.

### Effects of exercise training

Another important aspect to consider is the effect of sera conditioned by regular exercise *training* (chronic exercise) on cancer cell behaviour, once the effects of the last bout of exercise (i.e. acute exercise) have subsided. A series of early cross sectional studies demonstrated lower LNCaP prostate cancer cell growth and higher apoptosis when incubated with sera from regular exercisers as compared with inactive controls (Barnard et al. 2003, 2007; Tymchuk et al. 2002). However, there was a large difference in body mass index between the exercise and control groups (26 vs 38 kg/m^2^), which may explain much of the difference in serum-stimulated cancer cell behaviour (Barnard et al. 2007, 2003; Tymchuk et al. 2002). The same research group also identified that serum collected following a 2-week intervention combining regular aerobic exercise and a low-fat diet reduced cancer cell growth and increased apoptosis in vitro; however, the effects of exercise cannot be isolated from the effects of diet in these studies (Barnard et al. 2006; Ngo et al. 2003; Tymchuk et al. 2001). In addition, the post-intervention blood samples were drawn within a 24-h window following an acute bout of exercise (i.e. the final training session) and, therefore, the findings are likely to reflect transient acute effects rather than a training effect per se (Barnard et al. 2006; Ngo et al. 2003; Tymchuk et al. 2001). Similarly, a recent study by Schwappacher et al. (2020) reported reduced cell proliferation and increased apoptosis in human prostate and colon cancer cell lines that were incubated with post-training serum from patients with advanced stage cancer following 12 weeks of whole-body electromyostimulation to mimic resistance training. However, the authors also collected the post-training blood samples 1 h following the last training session, and so the effect of training cannot be isolated from the effects of the last bout of exercise. This is also the case for a recent pre-clinical study in a rodent model, which showed reduced prostate cancer AT-1 cell growth following incubation with serum collected 24-h after the final session of an 11-week training programme (Opoku-Acheampong et al. 2019), a time point where the acute effects of exercise may still be present (Baldelli et al. 2020; Kurgan et al. 2017).

Interestingly, a number of other recent publications presented no evidence of an effect of exercise training on cancer cell growth when post-training samples were collected greater than 1 day and no more than 7 days following the last exercise bout (Baldelli et al. 2020; Dethlefsen et al. 2016; Devin et al. 2019). This has been demonstrated using sera drawn from young healthy men on a prostate cancer cell line (Baldelli et al. 2020), serum drawn from men with colon cancer on colon cancer cell lines (Devin et al. 2019), and serum drawn from young healthy women (Baldelli et al. 2020) and women with breast cancer (Dethlefsen et al. 2016) on breast cancer cell lines. These studies have involved short (4 weeks) (Devin et al. 2019), short-to-medium (9 weeks) (Baldelli et al. 2020), and longer term (6-month) (Dethlefsen et al. 2016) exercise training interventions, and all of them have demonstrated physiological adaptations following training, including an increase in V̇O_2peak_/V̇O_2max_ (Baldelli et al. 2020; Dethlefsen et al. 2016; Devin et al. 2019). Therefore, the lack of effect on cancer cell behaviour does not appear to be explained by an insufficient training stimulus per se. On the other hand, it is worth noting that these studies have either been conducted in healthy lean populations (Baldelli et al. 2020) or have shown only small (Devin et al. 2019) or no (Dethlefsen et al. 2016) changes in body mass or body composition in overweight cancer patient populations following training. Longer term exercise interventions, particularly involving a large volume of aerobic exercise, are likely to have positive impacts on body mass and body composition in overweight or obese individuals (Ohkawara et al. 2007; Slentz et al. 2004). From this perspective, it is worth highlighting that the study that applied a 6-month exercise intervention only required a single supervised exercise session per week and reported low levels of adherence (mean attendance 66%) (Dethlefsen et al. 2016). Reductions in body fatness are known to drive changes in the circulating plasma profile, including alterations in key proteins/hormones/metabolites implicated in oncogenesis (McTiernan 2008). Thus, future studies should address the question of whether exercise training with and without weight loss results in different effects with regard to serum-stimulated cancer cell behaviour.

Another interesting question is whether the acute effects of exercise on cancer cell behaviour are still apparent after a period of regular exercise training. This is important because exercise training can diminish key skeletal-muscle signalling events (e.g. activation of 5′ AMP-activated protein kinase (AMPK)) (McConell et al. 2005, 2021) and alter skeletal muscle metabolism at the same (whole-body) relative exercise intensity (e.g. %V̇O_2_max), and this may have an impact on subsequent serological responses. To date, only the study by Baldelli et al. (2020) has considered this question using an appropriate design. They reported that the magnitude of the effect of acute exercise on prostate cancer cell growth may be attenuated by regular training, i.e. cell proliferation may be suppressed to a lesser extent following acute exercise after a period of training. The acute exercise bout used in this study involved a mixture of incremental aerobic exercise and HIIE, but was performed to exhaustion both pre- and post-training, so the attenuated effect on prostate cancer cell growth does not appear to be driven by changes in fitness and, hence, relative exercise intensity (Baldelli et al. 2020). Further studies evaluating whether the effect of acute exercise on cancer cell growth is attenuated following training, and whether this correlates with alterations in the change in the serological profile during acute exercise, may provide key information to help untangle the molecular mechanisms underlying this phenomenon.

## Acute exercise-conditioned serum and cancer cell growth: signalling and molecular mechanisms

**Fig. 2:**
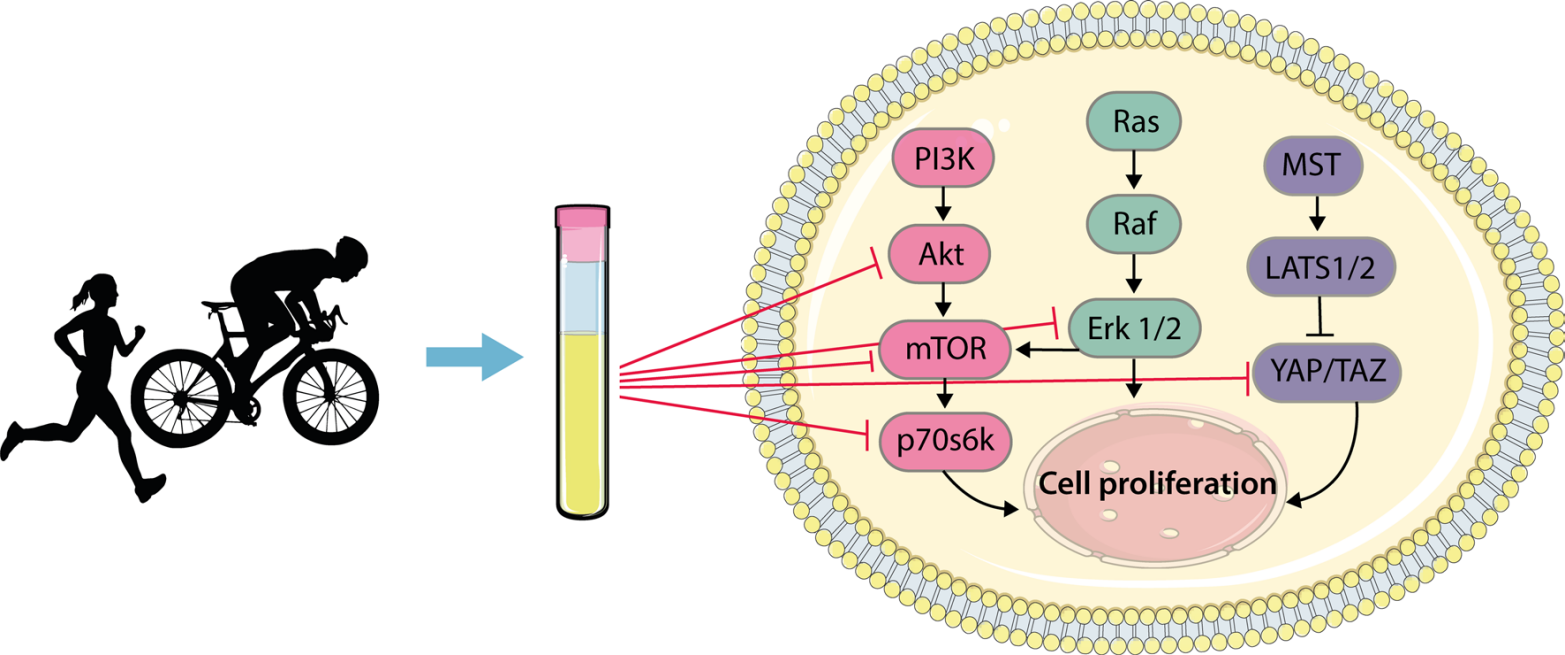
Acute-exercise-conditioned serum inhibits signalling pathways involved in cell proliferation. Increased proliferation in cancer cells is underpinned by mutations in highly conserved signalling networks that are involved in cell growth and division. Exposure of cancer cells to acute-exercise-conditioned serum has been shown to alter phosphorylation of proteins in these signalling pathways in a way that reduces cell proliferation. For example, exercise-conditioned serum was shown to reduce phosphorylation of Akt, mTOR, p70s6k and Erk 1/2 in human lung cancer cells (22). In addition, post-exercise serum has been suggested to support the Hippo tumour suppressor pathway (which involves activation of MST and LATS1/2) by inhibiting YAP/TAZ in human breast cancer cells (18); although, further studies are required to determine this since similar results were not observed in other cancer cell types (e.g. colon cancer cells (18)). Figure adapted from Kurgen et al. (2017) and reproduced under an open access Creative Common CC BY license

The increased rate of proliferation that is a hallmark of cancer cells is underpinned by multiple local genetic mutations in highly conserved signalling networks that, in healthy cells, regulate cell growth and division in response to both intracellular and extracellular stimuli (Fig. 2 (Shaw and Cantley 2006)). For example, in skeletal muscle cells, mTOR, and its upstream activators (e.g. PI3K, Akt, Ras) and downstream targets (e.g. p70s6k etc.), are responsive to both acute exercise (mechanical loading) and feeding (amino acids) and regulate protein synthesis and growth (Hodson and Philp 2019). The proteins in this pathway are also commonly dysregulated, and hence hyperactive, in a large proportion of different cancer types (Shaw and Cantley 2006). Kurgan et al. (2017) demonstrated that exposure to acute exercise-conditioned serum reduced phosphorylation of Akt, Erk 1/2, mTOR and p70S6K in human lung cancer cell lines, which was observed using serum obtained up to 24 h post-exercise. This may, in part, explain the effects of acute exercise sera on lung cancer cell growth. Whether this pathway is also suppressed within other cancer cell types is plausible, but requires investigation.

Two studies have also investigated the effect of post-exercise serum on the activation state of the Hippo tumour suppressor pathway (Fig. 2; Baldelli et al. 2020; Dethlefsen et al. 2017a), which is commonly *inactivated* in most cancers (Sebio and Lenz 2015; Serrano et al. 2013). This pathway involves a kinase cascade from the tumour suppressors mammalian Mst1 and Mst2 to the oncoproteins YAP and TAZ, which are transcriptional coactivators of target genes involved in cell growth and survival (Fig. 2) (Pan 2010). Following the activation of the Hippo pathway, which can be achieved through ligand binding to G protein-coupled receptors (e.g., β-adrenergic receptors) (Yu et al. 2012), Mst1/2 is phosphorylated and activates LATS1/2, which in turn phosphorylates YAP/TAZ, resulting in the inhibition and degradation of YAP/TAZ in the cytosol (Han 2019). However, the data from these studies are far from conclusive. Dethlefsen et al. (2017a) provided some evidence of increased phosphorylation, and thus inhibition, of the YAP protein, and reduced expression of its downstream target genes following incubation of breast cancer cells with post-exercise serum. However, the increase in YAP phosphorylation was not statistically significant across all the time points assessed within the assay and was inconsistent across two different human breast cancer cell lines (MCF-7 and MDA-MB-231 cells). Similarly, Baldelli et al. (2020) were unable to demonstrate a consistent suppression of Hippo-related signalling following incubation of cells with post-exercise serum, with increased YAP phosphorylation observed at one of six post-exercise time points in prostate cancer LNCaP cells, and no increase observed in triple-negative breast cancer MDA-MB-231 cells, despite consistent suppression of cell growth at all time points in both human cell lines. Taken together, it remains unclear whether activation of the Hippo pathway contributes to the anti-oncogenic effects of acute exercise (Orange et al. 2020).

The concentration of thousands of humoral factors are altered by acute exercise (Contrepois et al. 2020; Guseh et al. 2020) and the exact factor(s) responsible for the suppression of growth signalling and the anti-oncogenic effects of acute-exercise-conditioned sera remains to be determined. To investigate the role that exercise-induced epinephrine and norepinephrine play in cancer cells, Dethlefsen et al. (2017a) supplemented post-exercise serum with an ß-adrenergic signalling inhibitor (propranolol) prior to treatment of human breast cancer cells. They showed that this ablated the suppression of breast cancer cell growth in vitro, as well as tumour growth in mice following implantation with the treated cells. However, given that circulating epinephrine and norepinephrine concentrations return to basal levels within 3-h post moderate- and high-intensity exercise (Williams et al. 2013), this would not explain the suppression of cancer cell growth using serum obtained 24-h post exercise that was observed in some studies (Baldelli et al. 2020; Kurgan et al. 2017), unless downstream priming effects are involved.

A number of others have hypothesised that cytokines and other peptides released from contracting skeletal muscle during exercise (i.e. myokines) may play an important role in the effect of exercise sera on cancer cells (Hojman et al. 2018). However, to date, few myokines have been systematically investigated in the context of acute-exercise-mediated suppression of cancer cell growth. Interleukin 6 (IL-6) is the most widely researched myokine, and studies have consistently shown an exercise-induced increase in IL-6 mRNA within the contracting muscle, as well as increased levels of IL-6 in the circulation (Fischer 2006; Pedersen and Febbraio 2008; Ruffino et al. 2016). Directly stimulating oestrogen-receptor-positive breast cancer cells with IL-6 has been shown to reduce cell growth in vitro (Dethlefsen et al. 2013)*.* In addition, IL-6 can elicit both pro-inflammatory and anti-inflammatory processes, which likely depends on the cell source and whether IL-6 acts through classic or trans-signalling (Rose-John 2012). IL-6 signalling is initiated by binding of IL-6 to the IL-6 receptor α (IL-6Rα) and subsequent binding to the ubiquitously expressed receptor subunit glycoprotein 130 (gp130). IL-6Rα exists in a transmembrane and soluble form; binding of IL-6 to membrane bound IL-6Rα leads to anti-inflammatory classic signalling; whereas, binding of IL-6 to soluble IL-6Rα promotes pro-inflammatory trans-signalling (Reeh et al. 2019). A study using a mouse model with conditional expression of the IL6 gene showed that IL-6 derived from adipocytes increased macrophage infiltration of adipose tissue; whereas, IL-6 derived from myocytes and myeloid cells suppressed macrophage infiltration (Han et al. 2020). This opposing action of IL-6 was associated with a switch from trans-signalling to classic signalling (Han et al. 2020). Thus, as well as having a direct role in suppressing cancer growth, as evidenced by studies with oestrogen-receptor-positive breast cancer cells (Dethlefsen et al. 2013), IL-6 released from skeletal muscle during exercise may also play an indirect role through the induction of an anti-inflammatory environment. In further support of this notion, evidence exists demonstrating that IL-6 signalling stimulates the release of anti-inflammatory cytokines IL-10 and IL-1ra from monocytes and macrophages, leading to elevated levels of these cytokines in serum (Gleeson et al. 2011; Steensberg et al. 2003).

Aoi et al. (2013) demonstrated that the secreted protein acidic-rich in cysteine (SPARC) is also released from skeletal muscle into the circulation following 30 min of aerobic exercise in both mice and humans. They showed that SPARC induces a reduction in growth in a human colon cancer cell line in vitro and that regular injection of a low dose of SPARC into mice (to mimic the rise during acute exercise) reduced formation of colon cancer lesions (Aoi et al. 2013). A similar reduction in the formation of colon cancer lesions was reported with regular exercise in wild-type but not in SPARC-null mice (Aoi et al. 2013). Hojman et al. (2011) conducted a systematic investigation of the effects of IL-10 and IL-11, oncostatin M (OSM) and GDF5 on cancer cell behaviour using a mouse model. Of this panel of myokines, their data suggested that IL-10 and OSM are possible candidates mediating the effects of exercise on (breast) cancer cell proliferation. However, it should be noted that, since both of these studies are based on animal data, whether they are directly translatable to humans is unclear (Mak et al. 2014). Indeed, both SPARC and OSM were also associated with increased apoptosis and caspase activity in these animal studies (Aoi et al. 2013; Hojman et al. 2011) and current human data suggests that apoptosis is not increased in cancer cells treated with exercise-condition serum (Devin et al. 2019; Rundqvist et al. 2013). A range of other myokines are purported to have anti-carcinogenic effects, including irisin (Gannon et al. 2015) and decorin (Araki et al. 2009; Soria-Valles et al. 2014), but their relevance in humans in the context of the anti-oncogenic effects of acute exercise is yet to be demonstrated.

There are also several other plausible mechanisms to explain the anti-carcinogenic effects of exercise-conditioned serum that, to our knowledge, have not been investigated in the context of exercise and cancer. For example, IL-6 release from skeletal muscle during exercise is known to act in an endocrine manner to stimulate lipolysis and fatty acid oxidation in adipose tissue through the activation of AMPK (Kelly et al. 2004). AMPK is known to inhibit mTOR and its downstream effectors, such as p70s6k, through a tuberous sclerosis complex 2 (TSC2)‐dependent or ‐independent mechanism (Bolster et al. 2002; Kawaguchi et al. 2015). As mentioned above, mTOR signalling is dysregulated in many cancers, promoting the cell proliferation and metabolism that contribute to tumour initiation and progression (Shaw and Cantley 2006). Whether muscle-derived IL-6 can similarly activate AMPK in distant tissues other than adipocytes, such as aberrant or dysplastic cells, and subsequently suppress mTOR activity has not been systematically investigated.

Taken together, the signalling pathways that are altered by acute-exercise serum, and the serum components responsible for the potential anti-cancer properties, remain largely unclear, demonstrating a need for further research in this area. It is likely that the mechanisms will be nuanced and different depending on a range of factors, including the cancer type, the population and the exercise characteristics. It is also unlikely to be explained by the effect of a single protein or metabolite, but instead probably reflects the synergistic or competitive interactions of multiple serological changes that occur during and following exercise (although, of course, this remains speculative).

A review of the role of the immune system in the impact of exercise on cancer cell biology is out of the scope of this review (interested readers are directed to a recent review of this topic (Koelwyn et al. 2015)), but it is also interesting to note that, in addition to the potential direct effects of the immune system on the tumour itself, exercise-dependent proteomic factors within plasma can have powerful effects on circulating immune cells. For example, these factors may prime circulating immune cells to develop specific phenotypes following migration into tissues and differentiation, ultimately impacting their function within tissues (Idorn and Hojman 2016). As an example, circulating adrenaline is involved in the circulatory deployment of natural killer T-cells, whilst IL-6 regulates their distribution and activation, during and following acute exercise (Pedersen et al. 2016). Thus, exercise-stimulated serological changes are involved in both direct and indirect mechanisms that could impact upon tumorigenesis and cell growth and development in vivo.

## Physiological relevance and limitations of in vitro cancer models

It has been hypothesised that cumulative, small suppressive effects of acute exercise on cancer cell growth, when exercise is performed regularly over several weeks and months, would have marked effects on tumour growth in vivo (Dethlefsen et al. 2016, 2017b). Yet, it is reasonable to ask: what is the relevance of the effects observed in vitro for tumours forming and developing in the intact physiological system, and are they likely to have any direct consequences for cancer prevention and therapy? Indeed, the sceptic could draw parallels with circulating anabolic hormones and resistance-exercise-induced muscle growth: the serum concentration of several anabolic hormones, including testosterone, insulin-like growth factor 1, and human growth hormone, are increased following acute resistance exercise (Morton et al. 2016; West et al. 2009, 2010) and they potentiate myocellular growth in vitro (Jacquemin et al. 2004; Sculthorpe et al. 2012; Serra et al. 2011). However, in humans they have now been shown to have limited (if any) impact on the regulation of protein dynamics and changes in muscle hypertrophy in response to resistance training in vivo (Morton et al. 2016).

It is first important to consider how representative the in vitro systems are of the physiological scenario. For example, an important question is whether a tumour growing or developing in vivo will be exposed to the active component(s) of serum that exerts the suppressive effect on growth (as is the case in vitro). In vivo*,* this will depend on a combination of factors, including the level of vascularisation and perfusion to the tumour and whether the active proteins or metabolites can easily cross the endothelial barrier and penetrate into the interstitial fluid bathing the tumour. From the latter perspective, tumour vessels are known to be more permeable than healthy vessels (Chung et al. 2010) but, nevertheless, the pathway of trans-endothelial transport is likely to depend on the size of the anti-carcinogenic protein(s) or metabolite(s). Small molecules (< 3 nm, e.g. glucose) are able to diffuse easily through inter-endothelial junctions, but larger molecules (> 3 nm, e.g. hormones) require transport via receptor-mediated or other transcellular pathways (Mehta and Malik 2006). At the same time, tumour vascular networks are heterogeneous, and often immature and dysfunctional, leading to highly variable tumour blood flow and perfusion across whole tumours, or within different regions within a single tumour (Chung et al. 2010; Hughes et al. 2019; Schumacher et al. 2020). Poor perfusion of the tumour microenvironment can have a direct impact on the delivery of nutrients [e.g. creating areas of hypoxia (Hughes et al. 2019)] and, by extension, is also likely to mean that ‘exposure’ of the tumour to anti-oncogenic serological components is either low or may be completely absent. Indeed, tumours with a dysfunctional vascular tree and poor perfusion are known to be less responsive to systemic pharmacotherapies (Gillies et al. 1999). It is important to note, however, that during exercise there is enhanced blood flow to the tumour and decreased tumour hypoxia (McCullough et al. 2014), suggesting that exercise may increase the delivery of circulating factors to the tumour. Irrespective, it is reasonable to speculate that the acute effects of exercise in vivo are likely to be variable and dependent on the specific characteristics of the tumour.

There are several other limitations of in vitro cancer models. Notably, with the exception of two recent small studies (Baldelli et al. 2020; De Santi et al. 2019), the majority of work examining the effect of exercise-conditioned media has been conducted by using 2D monocultures of tumour cells grown on flat plastic surfaces. This does not fully reflect the in vivo physiological scenario given that key aspects of the tissue architecture and tumour microenvironment, including extracellular matrix, cell-to-cell contacts, fibroblasts and other tumour-associated stromal cells, are absent in such conditions (Kapałczyńska et al. 2018). It is also important to consider that only limited work has investigated the effect of exercise-conditioned sera on tumour cell migratory potential and invasiveness. Although the rate of tumour proliferation is an important measure, the rate of tumour growth and its metastatic potential do not always correlate. Indeed, in some cases, cancerous cells can invade and metastasise before or without growing much at the primary site (Dasgupta et al. 2017; Friberg and Nystrom 2015). The application of 3D assays, co-culture systems and/or the use of ‘scaffolds’ mimicking the extracellular matrix, will be necessary to overcome some of these limitations (Kapałczyńska et al. 2018) and will provide a more physiologically relevant examination of the effects of exercise-conditioned medium.

Another important limitation of the currently available in vitro studies is that they all employed established tumour cell lines. These cell lines might fail to predict in vivo tumour responses because of their restricted capacity to recapitulate inter-tumour and intra-tumour heterogeneity even when derived from the same tissue. In addition, the studies conducted to date have only demonstrated that exercise-conditioned sera reduce growth of already transformed cancer cells and do not permit to determine whether serological changes induced by exercise can inhibit the transformation of healthy or aberrant cells into cancerous cells.

Finally, it should also be noted that the suppressive effect on viability / growth only becomes *detectable* when the cancer cells are incubated with post-exercise serum in vitro for 24–96 h (or longer for clonogenic assays) (Baldelli et al. 2020; Dethlefsen et al. 2016, 2017a; Devin et al. 2019; Hwang et al. 2020; Kurgan et al. 2017; Rundqvist et al. 2013). An important translational consideration here is that the serum that the cancer cells are incubated with represents a ‘snapshot’ of the serological profile at one brief time point during a rapidly changing post-exercise period. During the in vitro assay, the cancer cells are continuously exposed to the serum and so the suppressive effect is presumably active (stimulated) throughout the duration of the assay (24–96 h). Of course, in the in vivo physiological system, a tumour will never be exposed for a prolonged period of time to post-exercise serum within a ‘snapshot’ in time, but rather, it will be exposed to the serological profile as it transitions through a period of disrupted homeostasis during exercise and as it recovers back to ‘baseline’ in the hours after exercise. On this basis, it is reasonable to question whether in vitro findings on the effect of exercise-conditioned serum on cancer cell behaviour are ever likely to be truly reflective of the in vivo scenario. Two key questions concerning physiological relevance arise from this reasoning; first, how long does the anti-carcinogenic effect of serum last into the post-exercise period? As discussed above, the current data on this are somewhat ambiguous, with some studies reporting that the effect is short-lived and lost in serum collected 2 h post-exercise (Devin et al. 2019), and others detecting effects of serum collected up to 24 h post-exercise (Baldelli et al. 2020; Kurgan et al. 2017). A clear answer to this question, and how the time-course of the effect might be impacted by the characteristics of the exercise bout, such as relative exercise intensity, duration and mode, is crucial, as it will provide a clearer indication of the likely duration of exposure of a tumour to the anti-carcinogenic mediators in vivo and it will allow clearer inferences on the likely relevance of the effects observed in vitro. Second, it will also be important to determine whether the alterations in cancer cell proliferation require continual stimulation with the active component(s) of the post-exercise serum or whether a brief, transient exposure results in downstream signalling alterations that affect proliferation even if the ‘stimulator’ is removed (i.e. a priming effect). From this perspective, it will be interesting for future research to perform (epi)genomic/transcriptomic profiling of cancer cells exposed to post-exercise serum to investigate whether priming effects may be involved.

## Conclusions

**Fig. 3:**
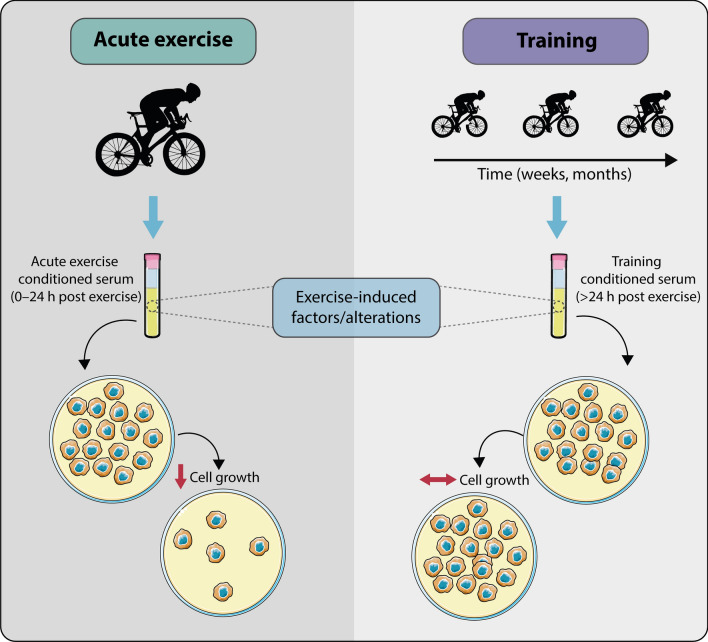
Summary of the effect of acute-exercise- vs chronic-exercise-conditioned serum on human cancer cell biology. In humans, acute exercise serum (collected 0–24 h post exercise) induces serological changes that suppress cancer cell growth but that do not appear to induce apoptosis in vitro. In comparison, there appears to be no effect of exercise-training-conditioned serum (collected > 24 h post chronic exercise, when the acute effects of the last bout of exercise have subsided) on cancer cell proliferation

A number of tentative conclusions can be drawn from the current review (summarised in Fig. 3). First, in humans, acute exercise appears to be associated with serological changes that suppress cancer cell growth but do not induce apoptosis in vitro. It remains unclear how long after exercise cessation the anti-growth effect persists; it is almost certainly observed immediately post-exercise with a variety of aerobic and HIIE protocols, but some studies report that the effect is lost soon after exercise and some have observed the effect for up to 24 h post-exercise (Table 1). Importantly, there appears to be no effect of exercise training on serum-stimulated cancer cell proliferation when blood samples are taken at a time point when the acute effects of the last bout of exercise have subsided (i.e. > 24 h post exercise). Taken together, these findings suggest that the serological effects of exercise may be an important mechanism underlying the beneficial effects of physical activity and exercise on cancer risk and outcome. The presence of an acute but not a training effect may have important practical implications for exercise prescription for cancer prevention and treatment, suggesting that a high frequency of exercise may be important to maximise the effects.

**Fig. 4:**
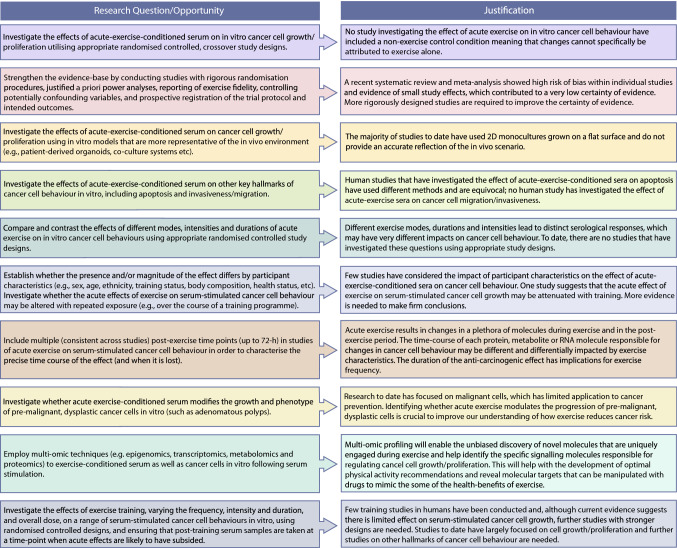
A roadmap of key research opportunities on exercise-conditioned serum and cancer cell behaviour

However, in many ways, our understanding in this area is in its infancy and there are number of important questions that required further investigation (summarised in Fig. 4). Applying more rigorous study designs to systematically comparing the effects of different exercise characteristics, particularly intensity, duration and mode, on cancer cell behaviour, will provide important information from the perspective of exercise prescription, but may also help to elucidate the underpinning molecular mechanisms, for example by comparing and contrasting exercise bouts that are effective and ineffective. At the same time, it will be important to clarify whether the acute effects of exercise are attenuated with repeated bouts, particularly given that the effects of training (without weight loss) appear to be limited. Perhaps most importantly, a key area for future research should be to characterise the effect of exercise-conditioned serum on in vitro cancer cell models that are more representative of the in vivo environment (e.g. patient-derived organoids, co-culture systems, pre-malignant polyps etc.). It is also reasonable to expect that the population from which the sera is derived may modify the subsequent responses to exercise. Indeed, it has recently been shown that age-associated changes in the serological environment can encourage tumour aggressiveness and metastasis (Gomes et al. 2020). Thus, characterising the effects of exercise on cancer cell behaviour in a range of different populations will be key. An overarching aim, encompassing each of these questions, should be to provide a precise time course of the responses, with the addition of multi-omic profiling applied in synergy, to help identify the active ingredients of exercise-conditioned sera and the molecular signalling pathways in cancer cells underlying the phenotypic effects. Answering these questions will bring together expertise in exercise physiology and cancer biology, ultimately improving our understanding of the relationship between physical activity and cancer to allow us to optimise recommendations for cancer prevention, treatment and survivorship, and identify novel therapeutic targets.
